# (10*Z*)-4*H*,5*H*,6*H*,7*H*,8*H*,9*H*-Cyclo­deca­[*d*][1,2,3]selena­diazole

**DOI:** 10.1107/S2414314624011076

**Published:** 2024-11-19

**Authors:** Dieter Schollmeyer, Heiner Detert

**Affiliations:** a University of Mainz, Department of Chemistry, Duesbergweg 10-14, 55099 Mainz, Germany; Goethe-Universität Frankfurt, Germany

**Keywords:** crystal structure, heterocycle, medium-sized ring, selenium

## Abstract

The title compound, C_10_H_14_N_2_Se, was prepared from a semicarbazone and selenium dioxide. The planes of the heterocycle and the *cis* double bond are almost mutually orthogonal and the hexa­methyl­ene tether is nearly strain-free.

## Abstract 2

In the title compound, which was prepared from a semicarbazone and selenium dioxide, the planes of the heterocycle and the *cis* double bond are almost mutually orthogonal and the hexa­methyl­ene tether is nearly strain-free.

## Graphical abstract

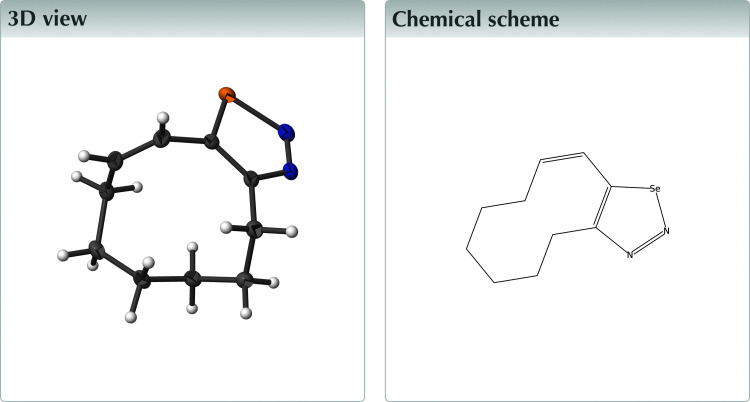

## Structure description

**Figure 1:**
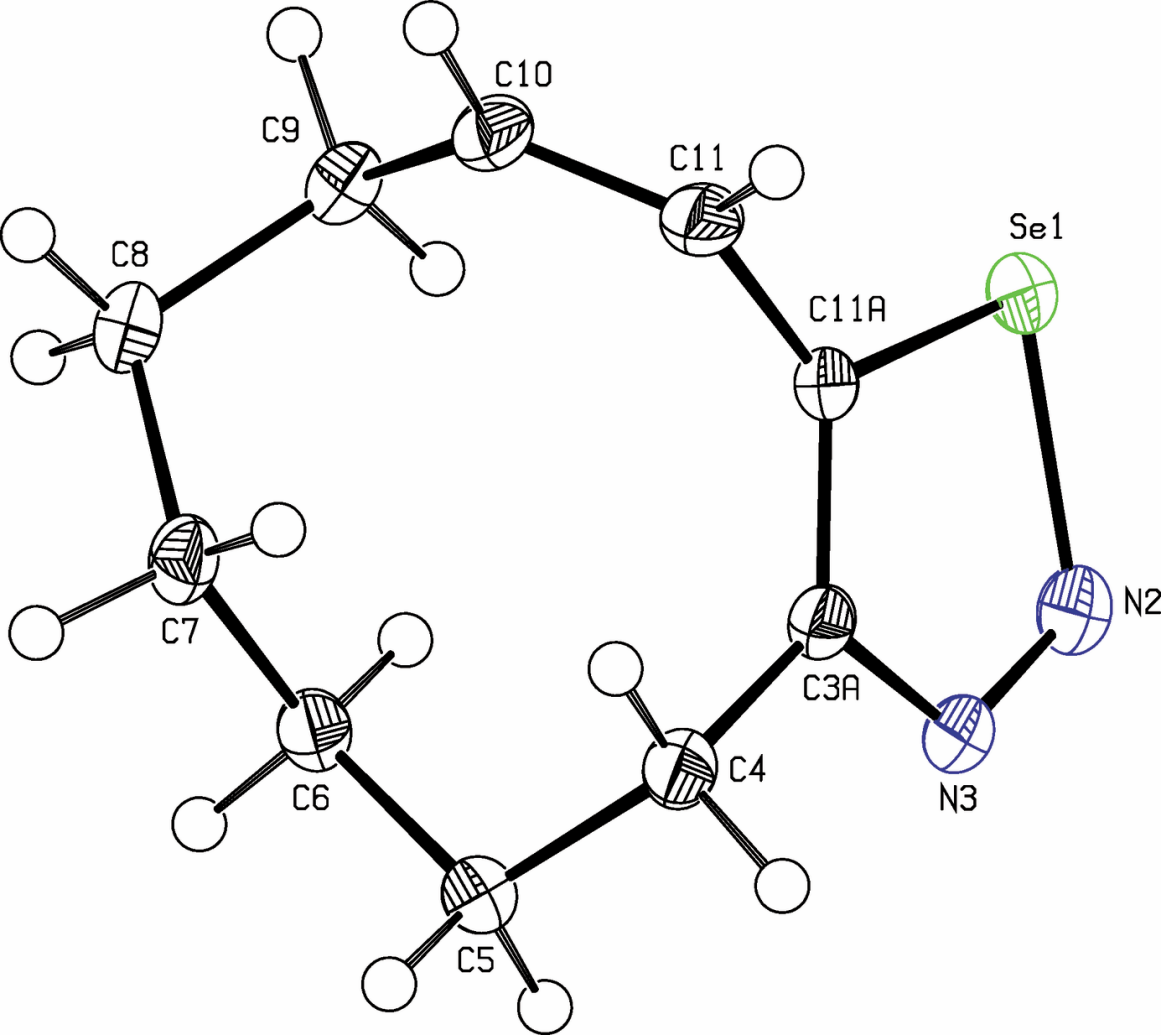
View of the title compound. Displacement ellipsoids are drawn at the 50% probability level.

**Figure 2:**
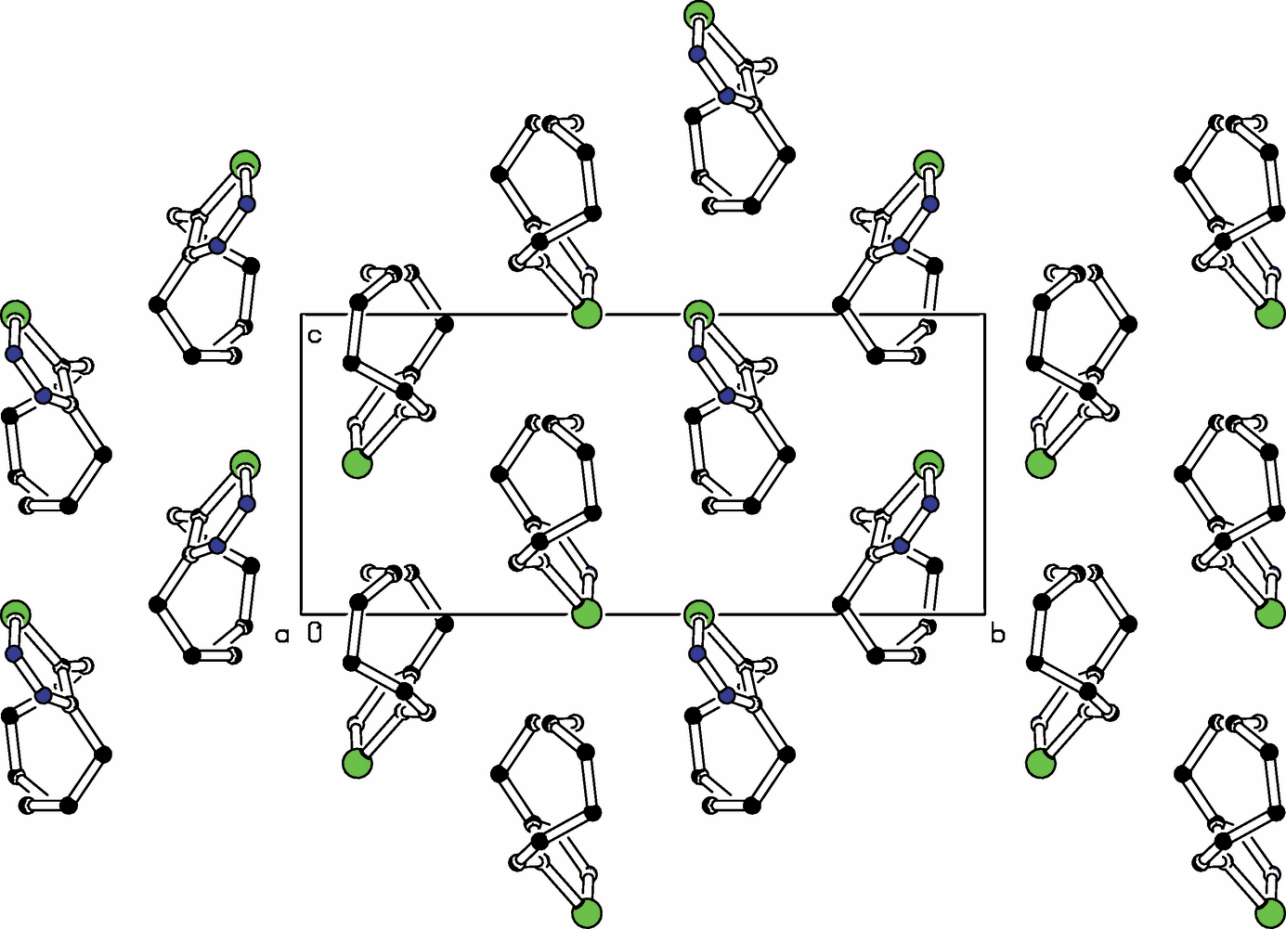
Part of the packing diagram. View along the *a* axis.

The title compound, C_10_H_14_N_2_Se (Fig. 1 ▸), was prepared as part of a project focusing on medium-sized cyclo­alkynes (Bissinger *et al.*, 1988 ▸; Detert & Meier, 1997 ▸). Thermolysis of 1,2,3-selena­diazo­les is an advantageous route to strained cyclo­alkynes. They are prepared by oxidation of semicarbazones with selenium dioxide (Lalezari *et al.*, 1972 ▸). Selenious acid oxidized *Z*-cyclo­dec-3-enone semicarbazone to a mixture of the title compound (63%) and the homoconjugated (5*Z*)-isomer. In the crystal, the mol­ecules are arranged in layers parallel to the *ac* plane. Within a layer, all mol­ecules adopt the same orientation, while in the neighbouring layers, the orientation of the mol­ecules is inverted. The selena­diazole ring is essentially planar with an r.m.s. deviation of 0.002 (2) Å. In addition, the connecting atoms of the aliphatic tether are coplanar, C4 lies only 0.044 (2) Å above and C11 − 0.012 (2) Å below the selena­diazole plane. A negligible torsion angle [0.02 (4)°] twists the double bond (C10=C11) but the dihedral angle of 88.56 (15)° between the heterocycle and *cis*-olefin disrupts the π-conjugation. The hexa­methyl­ene chain shows a strain-free staggered arrangement. The packing is shown in Fig. 2 ▸.

## Synthesis and crystallization

The title compound was prepared in 63% yield from the semicarbazone of (3*Z*)-cyclo­decenone. The required ketone appeared in 10% yield upon selenious acid catalyzed hydrolysis/isomerization of (2*Z*)-cyclo­decenone semicarbazone (Whitham & Zaidlewicz, 1972 ▸; Hirano *et al.*, 1974 ▸). Selenium dioxide (2 mmol) was added to 0.5 mmol of the semicarbazone in 10 ml of 1,4-dioxane. After 3 days stirring, the solvent was evaporated, the slurry was mixed with toluene, washed with water, dried (MgSO_4_) and the compound isolated *via* chromatography with toluene/ethyl acetate on silica gel. Yield: 63% of yellowish crystals with the typical fetid odor of selena­diazo­les. NMR analysis at 298 K gave clear signals for the olefinic subunit but broad signals for the methyl­ene chain, indicating constricted conformational inter­conversions on the NMR time scale. Only at very low temperatures did the diastereotopic protons *e.g.* at C-4 gave separate signals of good resolution. M.p. = 315 K. ^1^H NMR (400 MHz, CDCl_3_, 293 K): 6.27 (*d*, 1 H, *J* = 11 Hz, H—C-11), 5.88 (*ddd*, 1 H, *J* = *J*′ = 11 Hz, *J*′′ = 5.5 Hz, H—C-10), 3.05 (*bs*, 2 H), 2.96 (*bs*, 2 H), 1.74 (*bs*, 2 H), 1.48 (*m*, 4 H), 0.99 (*bs*, 2 H); (400 MHz, CDCl_3_, 228 K): ; (400 MHz, CDCl_3_, 228 K): 6.25 (*d*, 1 H, *J* = 11 Hz, H—C-11), 5.85 (*ddd*, 1 H, *J* = *J*′ = 11 Hz, *J*′′ = 5.5 Hz, HC-10), 3.18 (pseudo-*d*, 1 H, *J* = 14 Hz, H—CH-4), 2.78 (*ddd*, *J* = *J*′ = 14 Hz, *J*′′ = 4 Hz, HC—H-4), 1.95 (*m*, 3 H), 1.56 (*t*, *J* = 13 Hz), 1.42 (*m*, 1 H), 1.25 (*m*, 4 H), 0.58 (*m*, 3 H). ^13^C NMR (CDCl_3_): 159.5 (^2^*J*C—Se = 32 Hz, C-3a), 155.1 (^1^*J*C—Se = 130 Hz, C-11*a*), 138.9 (^2^*J*C—Se = 37 Hz, C-11), 119.9 (C-10), 26.8, 25.6, 24.9, 24.7, 20.9, 20.5 (C4 - C9). ^77^Se NMR (CDCl_3_): 221.2 UV–Vis (EtOH): 224 (2.93), 239 (3.49), 294 (3.41) nm (logɛ) IR (CDCl_3_): 3005, 2920, 2840, 1495, 1430, 1310, 1255, 1210 cm^−1^.

## Refinement

Crystal data, data collection and structure refinement details are summarized in Table 1 ▸.

## Supplementary Material

Crystal structure: contains datablock(s) I, global. DOI: 10.1107/S2414314624011076/bt4160sup1.cif

Structure factors: contains datablock(s) I. DOI: 10.1107/S2414314624011076/bt4160Isup2.hkl

Supporting information file. DOI: 10.1107/S2414314624011076/bt4160Isup3.cml

CCDC reference: 2402782

Additional supporting information:  crystallographic information; 3D view; checkCIF report

## Figures and Tables

**Table 1:** Experimental details

Crystal data	
Chemical formula	C_10_H_14_N_2_Se
*M*_r_	241.19
Crystal system, space group	Monoclinic, *P*2_1_/*n*
Temperature (K)	120
*a*, *b*, *c* (Å)	7.9646 (6), 17.0830 (15), 8.0572 (6)
β (°)	111.425 (6)
*V* (Å^3^)	1020.50 (15)
*Z*	4
Radiation type	Mo *K*α
μ (mm^−1^)	3.64
Crystal size (mm)	0.29 × 0.21 × 0.11

Data collection	
Diffractometer	Stoe *IPDS* 2T
Absorption correction	Integration [*X-RED32* (Stoe & Cie, 2020 ▸), absorption correction by Gaussian integration (Coppens, 1970 ▸)]
*T*_min_, *T*_max_	0.381, 0.697
No. of measured, independent and observed [*I* > 2σ(*I*)] reflections	5508, 2430, 2093
*R*_int_	0.020
(sin θ/λ)_max_ (Å^−1^)	0.658

Refinement	
*R*[*F*^2^ > 2σ(*F*^2^)], *wR*(*F*^2^), *S*	0.030, 0.067, 1.12
No. of reflections	2430
No. of parameters	118
H-atom treatment	H-atom parameters constrained
Δρ_max_, Δρ_min_ (e Å^−3^)	0.41, −0.40

Computer programs: *X-AREA WinXpose*, *Recipe* and *Integrate* (Stoe & Cie, 2020 ▸), *SHELXT2014* (Sheldrick, 2015*a* ▸), *SHELXL2019/2* (Sheldrick, 2015*b* ▸) and *PLATON* (Spek, 2020 ▸).

## full crystallographic data

### (10Z)-4H,5H,6H,7H,8H,9H-Cyclodeca[d][1,2,3]selenadiazole Crystal data

**Table:**

C_10_H_14_N_2_Se	*D*_x_ = 1.570 Mg m^−^^3^
*M**_r_* = 241.19	Melting point: 315 K
Monoclinic, *P*2_1_/*n*	Mo *K*α radiation, λ = 0.71073 Å
*a* = 7.9646 (6) Å	Cell parameters from 10316 reflections
*b* = 17.0830 (15) Å	θ = 2.4–28.4°
*c* = 8.0572 (6) Å	µ = 3.64 mm^−^^1^
β = 111.425 (6)°	*T* = 120 K
*V* = 1020.50 (15) Å^3^	Block, colorless
*Z* = 4	0.29 × 0.21 × 0.11 mm
*F*(000) = 488	

### (10Z)-4H,5H,6H,7H,8H,9H-Cyclodeca[d][1,2,3]selenadiazole Data collection

**Table:**

Stoe IPDS 2T diffractometer	2430 independent reflections
Radiation source: sealed X-ray tube, 12x0.4mm long-fine focus	2093 reflections with *I* > 2σ(*I*)
Detector resolution: 6.67 pixels mm^-1^	*R*_int_ = 0.020
rotation method, ω scans	θ_max_ = 27.9°, θ_min_ = 2.4°
Absorption correction: integration [X-Red32 (Stoe & Cie, 2020), absorption correction by Gaussian integration (Coppens, 1970)]	*h* = −7→10
*T*_min_ = 0.381, *T*_max_ = 0.697	*k* = −21→22
5508 measured reflections	*l* = −10→10

### (10Z)-4H,5H,6H,7H,8H,9H-Cyclodeca[d][1,2,3]selenadiazole Refinement

**Table:**

Refinement on *F*^2^	Primary atom site location: dual
Least-squares matrix: full	Hydrogen site location: inferred from neighbouring sites
*R*[*F*^2^ > 2σ(*F*^2^)] = 0.030	H-atom parameters constrained
*wR*(*F*^2^) = 0.067	*w* = 1/[σ^2^(*F*_o_^2^) + (0.0224*P*)^2^ + 1.2555*P*] where *P* = (*F*_o_^2^ + 2*F*_c_^2^)/3
*S* = 1.12	(Δ/σ)_max_ < 0.001
2430 reflections	Δρ_max_ = 0.41 e Å^−^^3^
118 parameters	Δρ_min_ = −0.40 e Å^−^^3^
0 restraints	

### (10Z)-4H,5H,6H,7H,8H,9H-Cyclodeca[d][1,2,3]selenadiazole Special details

**Table:**

Geometry. All esds (except the esd in the dihedral angle between two l.s. planes) are estimated using the full covariance matrix. The cell esds are taken into account individually in the estimation of esds in distances, angles and torsion angles; correlations between esds in cell parameters are only used when they are defined by crystal symmetry. An approximate (isotropic) treatment of cell esds is used for estimating esds involving l.s. planes.
Refinement. Hydrogen atoms were placed at calculated positions and were refined in the riding-model approximation with C_aromatic_–H = 0.95 Å or C_methylene_–H = 0.99 Å and with *U*_iso_(H) = 1.2 *U*_eq_(C).

### (10Z)-4H,5H,6H,7H,8H,9H-Cyclodeca[d][1,2,3]selenadiazole Fractional atomic coordinates and isotropic or equivalent isotropic displacement parameters (Å^2^)

**Table:**

	*x*	*y*	*z*	*U*_iso_*/*U*_eq_	
Se1	0.22155 (3)	0.41792 (2)	0.00291 (3)	0.02588 (8)	
N2	0.0679 (3)	0.42034 (14)	0.1321 (3)	0.0287 (5)	
N3	0.1303 (3)	0.37734 (13)	0.2705 (3)	0.0242 (4)	
C3A	0.2906 (3)	0.33871 (13)	0.3010 (3)	0.0200 (4)	
C4	0.3665 (3)	0.29010 (14)	0.4674 (3)	0.0223 (5)	
H4A	0.467261	0.257977	0.460615	0.027*	
H4B	0.271954	0.254034	0.474213	0.027*	
C5	0.4350 (3)	0.34033 (14)	0.6375 (3)	0.0227 (5)	
H5A	0.331482	0.368148	0.650443	0.027*	
H5B	0.486911	0.305438	0.741913	0.027*	
C6	0.5776 (3)	0.40050 (14)	0.6379 (3)	0.0223 (5)	
H6A	0.525551	0.434969	0.532727	0.027*	
H6B	0.606410	0.433562	0.745666	0.027*	
C7	0.7529 (3)	0.36533 (14)	0.6347 (3)	0.0231 (5)	
H7A	0.836267	0.356584	0.758932	0.028*	
H7B	0.725966	0.313698	0.574953	0.028*	
C8	0.8476 (3)	0.41614 (15)	0.5396 (3)	0.0235 (5)	
H8A	0.965934	0.392425	0.555708	0.028*	
H8B	0.870326	0.468471	0.596223	0.028*	
C9	0.7400 (3)	0.42610 (13)	0.3392 (3)	0.0217 (5)	
H9A	0.626221	0.454180	0.322631	0.026*	
H9B	0.810602	0.458548	0.286372	0.026*	
C10	0.6965 (3)	0.34927 (15)	0.2428 (3)	0.0233 (5)	
H10	0.794761	0.321303	0.231365	0.028*	
C11	0.5350 (3)	0.31628 (15)	0.1718 (3)	0.0235 (5)	
H11	0.527227	0.267070	0.114513	0.028*	
C11A	0.3666 (3)	0.35080 (14)	0.1757 (3)	0.0200 (4)	

### (10Z)-4H,5H,6H,7H,8H,9H-Cyclodeca[d][1,2,3]selenadiazole Atomic displacement parameters (Å^2^)

**Table:**

	*U*^11^	*U*^22^	*U*^33^	*U*^12^	*U*^13^	*U*^23^
Se1	0.01898 (12)	0.03822 (15)	0.01753 (12)	−0.00109 (10)	0.00322 (8)	0.00338 (10)
N2	0.0176 (9)	0.0410 (13)	0.0244 (10)	0.0014 (9)	0.0042 (8)	0.0008 (9)
N3	0.0153 (9)	0.0351 (12)	0.0216 (10)	−0.0003 (8)	0.0059 (8)	−0.0011 (8)
C3A	0.0165 (10)	0.0235 (12)	0.0198 (10)	−0.0040 (8)	0.0063 (8)	−0.0041 (9)
C4	0.0195 (11)	0.0246 (11)	0.0217 (11)	−0.0025 (9)	0.0061 (9)	0.0008 (9)
C5	0.0205 (11)	0.0279 (12)	0.0190 (11)	−0.0006 (9)	0.0063 (9)	0.0010 (9)
C6	0.0211 (11)	0.0243 (12)	0.0198 (11)	−0.0008 (9)	0.0054 (9)	−0.0015 (9)
C7	0.0169 (10)	0.0248 (12)	0.0232 (11)	0.0006 (9)	0.0021 (9)	0.0037 (9)
C8	0.0163 (10)	0.0250 (11)	0.0257 (11)	−0.0027 (9)	0.0035 (9)	−0.0019 (10)
C9	0.0168 (10)	0.0214 (12)	0.0264 (11)	−0.0005 (8)	0.0073 (9)	0.0014 (9)
C10	0.0180 (10)	0.0284 (12)	0.0256 (11)	0.0011 (9)	0.0105 (9)	−0.0028 (9)
C11	0.0229 (11)	0.0277 (12)	0.0229 (11)	−0.0014 (9)	0.0121 (9)	−0.0053 (9)
C11A	0.0168 (10)	0.0255 (12)	0.0164 (10)	−0.0046 (8)	0.0048 (8)	−0.0034 (9)

### (10Z)-4H,5H,6H,7H,8H,9H-Cyclodeca[d][1,2,3]selenadiazole Geometric parameters (Å, º)

**Table:**

Se1—C11A	1.847 (2)	C6—H6B	0.9900
Se1—N2	1.875 (2)	C7—C8	1.527 (3)
N2—N3	1.274 (3)	C7—H7A	0.9900
N3—C3A	1.377 (3)	C7—H7B	0.9900
C3A—C11A	1.370 (3)	C8—C9	1.536 (3)
C3A—C4	1.503 (3)	C8—H8A	0.9900
C4—C5	1.538 (3)	C8—H8B	0.9900
C4—H4A	0.9900	C9—C10	1.500 (3)
C4—H4B	0.9900	C9—H9A	0.9900
C5—C6	1.531 (3)	C9—H9B	0.9900
C5—H5A	0.9900	C10—C11	1.327 (3)
C5—H5B	0.9900	C10—H10	0.9500
C6—C7	1.529 (3)	C11—C11A	1.475 (3)
C6—H6A	0.9900	C11—H11	0.9500
			
C11A—Se1—N2	87.18 (9)	C6—C7—H7A	108.8
N3—N2—Se1	110.36 (16)	C8—C7—H7B	108.8
N2—N3—C3A	118.2 (2)	C6—C7—H7B	108.8
C11A—C3A—N3	115.3 (2)	H7A—C7—H7B	107.6
C11A—C3A—C4	126.8 (2)	C7—C8—C9	113.71 (19)
N3—C3A—C4	117.9 (2)	C7—C8—H8A	108.8
C3A—C4—C5	112.50 (19)	C9—C8—H8A	108.8
C3A—C4—H4A	109.1	C7—C8—H8B	108.8
C5—C4—H4A	109.1	C9—C8—H8B	108.8
C3A—C4—H4B	109.1	H8A—C8—H8B	107.7
C5—C4—H4B	109.1	C10—C9—C8	112.46 (19)
H4A—C4—H4B	107.8	C10—C9—H9A	109.1
C6—C5—C4	113.46 (19)	C8—C9—H9A	109.1
C6—C5—H5A	108.9	C10—C9—H9B	109.1
C4—C5—H5A	108.9	C8—C9—H9B	109.1
C6—C5—H5B	108.9	H9A—C9—H9B	107.8
C4—C5—H5B	108.9	C11—C10—C9	126.8 (2)
H5A—C5—H5B	107.7	C11—C10—H10	116.6
C7—C6—C5	114.7 (2)	C9—C10—H10	116.6
C7—C6—H6A	108.6	C10—C11—C11A	124.5 (2)
C5—C6—H6A	108.6	C10—C11—H11	117.8
C7—C6—H6B	108.6	C11A—C11—H11	117.8
C5—C6—H6B	108.6	C3A—C11A—C11	127.3 (2)
H6A—C6—H6B	107.6	C3A—C11A—Se1	109.01 (17)
C8—C7—C6	114.0 (2)	C11—C11A—Se1	123.72 (17)
C8—C7—H7A	108.8		
			
C11A—Se1—N2—N3	−0.04 (18)	C8—C9—C10—C11	110.4 (3)
Se1—N2—N3—C3A	0.2 (3)	C9—C10—C11—C11A	0.2 (4)
N2—N3—C3A—C11A	−0.3 (3)	N3—C3A—C11A—C11	−178.8 (2)
N2—N3—C3A—C4	178.1 (2)	C4—C3A—C11A—C11	2.9 (4)
C11A—C3A—C4—C5	108.5 (3)	N3—C3A—C11A—Se1	0.3 (3)
N3—C3A—C4—C5	−69.7 (3)	C4—C3A—C11A—Se1	−178.00 (18)
C3A—C4—C5—C6	−56.7 (3)	C10—C11—C11A—C3A	−92.0 (3)
C4—C5—C6—C7	−63.5 (3)	C10—C11—C11A—Se1	89.0 (3)
C5—C6—C7—C8	149.2 (2)	N2—Se1—C11A—C3A	−0.12 (17)
C6—C7—C8—C9	−65.1 (3)	N2—Se1—C11A—C11	179.0 (2)
C7—C8—C9—C10	−57.5 (3)		
